# A Method of Optimizing Weight Allocation in Data Integration Based on Q-Learning for Drug-Target Interaction Prediction

**DOI:** 10.3389/fcell.2022.794413

**Published:** 2022-03-04

**Authors:** Jiacheng Sun, You Lu, Linqian Cui, Qiming Fu, Hongjie Wu, Jianping Chen

**Affiliations:** ^1^ School of Electronic and Information Engineering, SuZhou University of Science and Technology, Suzhou, China; ^2^ Jiangsu Province Key Laboratory of Intelligent Building Energy Efficiency, Suzhou University of Science and Technology, Suzhou, China; ^3^ Suzhou Key Laboratory of Mobile Network Technology and Application, Suzhou University of Science and Technology, Suzhou, China; ^4^ School of Architecture and Urban Planning, Suzhou University of Science and Technology, Suzhou, China

**Keywords:** drug-target interactions, heterogeneous information fusion, q-learning, weight distribution, drug similarity, target similarity

## Abstract

Calculating and predicting drug-target interactions (DTIs) is a crucial step in the field of novel drug discovery. Nowadays, many models have improved the prediction performance of DTIs by fusing heterogeneous information, such as drug chemical structure and target protein sequence and so on. However, in the process of fusion, how to allocate the weight of heterogeneous information reasonably is a huge challenge. In this paper, we propose a model based on Q-learning algorithm and Neighborhood Regularized Logistic Matrix Factorization (QLNRLMF) to predict DTIs. First, we obtain three different drug-drug similarity matrices and three different target-target similarity matrices by using different similarity calculation methods based on heterogeneous data, including drug chemical structure, target protein sequence and drug-target interactions. Then, we initialize a set of weights for the drug-drug similarity matrices and target-target similarity matrices respectively, and optimize them through Q-learning algorithm. When the optimal weights are obtained, a new drug-drug similarity matrix and a new drug-drug similarity matrix are obtained by linear combination. Finally, the drug target interaction matrix, the new drug-drug similarity matrices and the target-target similarity matrices are used as inputs to the Neighborhood Regularized Logistic Matrix Factorization (NRLMF) model for DTIs. Compared with the existing six methods of NetLapRLS, BLM-NII, WNN-GIP, KBMF2K, CMF, and NRLMF, our proposed method has achieved better effect in the four benchmark datasets, including enzymes(E), nuclear receptors (NR), ion channels (IC) and G protein coupled receptors (GPCR).

## Introduction

Diseases are usually caused by defective proteins in the body or the functional structure of viral proteins. Effective drugs can combine well with these proteins, removing the original function and achieving the therapeutic effect (Ding et al., 2021). Previous research and development of novel drugs mainly relied on biochemical experiments, which are risky, expensive and time-consuming. In addition, protein-protein interactions play a key role in many biological processes (Guo et al., 2015), leading to the emergence of large-scale experimental data on genes and proteins, making drug discovery and repositioning in biomedical research more difficult (Ding et al., 2019a). The purpose of DTIs prediction is to identify potential novel drugs or novel targets for existing drugs, and provide a list of candidate drugs for drug discovery, thus greatly improving the efficiency of research and development and reducing the cost of experiments. In recent years, the research methods of DTIs prediction have been proposed successively. So far, a large number of associations known to date have been proved by previous experiments and stored in some public databases (Wang et al., 2021). Therefore, existing methods predict DTIs mainly based on a small number of experimentally validated interactions in existing databases, such as DrugBank (Wishart et al., 2008), KEGG DRUG (Kanehisa et al., 2012), and SuperTarget (Günther et al., 2008). Previous studies have shown that DTIs prediction based on experimental verification can effectively predict some novel interactions between drugs and targets, and the computational methods used to predict DTIs can significantly improve the efficiency of drug discovery.

In general, traditional computational methods proposed for DTIs prediction are mainly based on ligands and target-based (Liu et al., 2021). The ligand-based method usually compares the candidate ligand with the known interacting ligand of the target to determine the binding between them. However, the disadvantage is that the ligand-based method cannot be used for targets which have no or only a small amount of known binding ligands. The target-based (or docking simulation) method uses docking techniques to predict interactions between drug candidates and targets. Nevertheless, this method requires detailed structures. Not all proteins have structural information (Ding et al., 2020). For example, most targets of GPCRs (G protein coupled receptors) are unknown (Yan et al., 2019).

In order to solve the difficulties of traditional methods, chemical genomics methods have been successfully used for large-scale drug discovery and repositioning (Chen et al., 2018). The purpose of chemical genomics research is to integrate drug and target information into a unified framework, in order to identify potentially useful compounds (Ezzat et al., 2019). Chemical genomics methods are usually divided into ligand-based, target-based, and target-ligand, all of which are based on similarity between member proteins and targets. In fact, this significant similarity-based view of chemical genomics has made machine learning methods widely used in DTIs prediction tasks (Ding et al., 2017; Lo et al., 2018; Ding et al., 2019b; Ding et al., 2019c; Qu et al., 2019; Liu et al., 2020; Bagherian et al., 2021), where DTIs prediction is regarded as a binary classification problem, in which drug-target pairs are taken as examples, the internal chemical structure of the drug and the amino acid subsequence of the target are considered as features. Then, we can build a binary classification model through some classic classification methods, such as support vector machines (SVM), neural networks and nearest neighbors. Yamanishi et al. (Yamanishi et al., 2010) proposed a framework for supervised bipartite graph reasoning, and this framework could predict unknown DTIs based on chemical, genomic and pharmacological data. Tabei et al. (Tabei et al., 2012) used complex protein pairs represented by eigenvectors to correspond to chemical substructures and protein domains, and used logistic regression and SVM to establish a prediction model. Mei et al. (Mei et al., 2013a) improved the bipartite local model by considering novel drug candidates through the interaction profile of neighbors. In short, DTIs prediction based on machine learning comes down to a process of data collection, feature representation, similarity calculation and machine learning modeling. Although machine learning has achieved good results in DTIs prediction, there are still bottlenecks. For example, most drug relocation methods based on machine learning only use a single metric to evaluate the similarity between diseases and between drugs. In fact, the similarity of drugs/diseases is not only noisy, but also multimodal, which can be measured from different aspects (Yang et al., 2021). The fusion of multiple similarity measures can avoid the noise and extract effective features in individual similarity calculation, thus effectively improving the accuracy of DTIs prediction.

In order to overcome the bottleneck encountered by machine learning in DTIs prediction, improving the prediction performance of DTIs by integrating heterogeneous information related to drugs and targets has been extensively studied by the academic community. Among them, the challenge is how to extract and integrate such information (Wang et al., 2011). Wang et al. (Wang et al., 2011) proposed a kernel function method based on SVM predictor to integrate heterogeneous information sources to improve the accuracy of DTIs prediction. Yan et al. (Yan et al., 2019) proposed a method based on multi-kernel learning and clustering to integrate heterogeneous information sources related to multiple drugs and targets to improve the accuracy of DTIs prediction, in which the weight of linear weighting was obtained through interior point optimization algorithm (Byrd et al., 1999). (Ding et al., 2020) used six different features to characterize protein sequences in the experimental process of predicting the subcellular localization of proteins. These features are constructed into corresponding kernels and combined by optimizing the weight of these kernels by Kernel Target Alignment-based Multiple Kernel Learning (KTA-MKL) (Ding et al., 2020). (Zhou et al., 2021) built a multi-kernel learning model with Hilbert-Schmidt independence criterion (HSIC) to obtain optimal weights for vairous features, and identified ncRNA subcellular localization by using the graph regularization k local hyperplane distance nearest neighbor model**.** Although the above methods have achieved good results, there are still some problems. For example, Wang et al. (Wang et al., 2011) found that the experimental effect of fusion of three information sources was inferior to that of fusion of two information sources in the process of fusion, and the reason may be that drug targets for the contributions to the similarity of DTIs prediction is different, and the integration of equal-weight information cannot reflect the difference of contribution ratio of heterogeneous information, DTIs prediction accuracy improved range is limited. To solve the above problems, we used Q-learning algorithm to reasonably allocate weights in the process of heterogeneous information integration, and realized DTIs prediction based on the NRLMF model proposed by Liu et al. (Liu et al., 2016). Finally, our method QLNRLMF achieved good results on four benchmark datasets.

The main contributions of this paper are summarized as follows:

- According to three heterogeneous data sources, including drug chemical structure, target protein sequence and drug-target interaction, we obtain three drug-drug similarity matrices and three target-target similarity matrices using different similarity calculation methods.
- We use Q-learning algorithm to reasonably allocate weight to the similarity matrices obtained above, and then perform linear weighted integration after obtaining the optimal weight.
- We perform DTIs prediction experiments on four benchmark datasets. Experimental results show that the DTIs prediction accuracy of QLNRLMF model is better to several advanced comparison models

## Materials and Methods

### Problem Description

In this paper, the drug set is defined as D = ${\left\{d_{i}\right\}}_{i=1}^{m}$ , and the target set is defined as T = ${\left\{t_{j}\right\}}_{j=1}^{n}$ , where m and n respectively represent the number of drugs and the number of targets. The interaction between the drug and the target is defined as Y ∈ $R^{m\;\times \;n}$ , which is composed of drug $d_{i}$ (1 ≤ *i* ≤ *m*) and target $t_{j}$ (1 ≤ *j* ≤ *n*), the matrix Y ∈ $R^{m\;\times \;n}$ is defined as Eq. 1.

$$Y_{ij}=\{\;\;\;\begin{array}{cc} 1 & Drug\;d_{i}\;is\;related\;to\;Target\;t_{j}\\ 0 & Drug\;d_{i}\;is\;not\;related\;to\;Target\;t_{j} \end{array}$$ (1)

We take each drug-target pair in Y as a sample, and divide equally all drug-target pairs in Y into ten pieces by random seed shuffled. Then we select one sample as the test set in turn, and the remaining nine samples as the training set. At the same time, we obtain all the samples in the test set corresponding to the subscripts in Y and assign all the corresponding positions to 0, and a new drug and target interaction $Y_{train}$ ∈ $R^{m\;\times \;n}$ is obtained. The purpose of our experiment is to predict these unknown pairs of interactions labeled 0.

### Data Preparation

#### Chemical Data

Yamanishi et al. (Yamanishi et al., 2008) obtained the chemical structure of the compound from the drugs and compounds section of the KEGG ligand database (Kanehisa et al., 2007). The chemical structure similarity between drugs is obtained by the SIMCOMP algorithm (Hattori et al., 2003), and the similarity score is calculated according to the number of common substructures between two compounds. In this paper, the chemical structure similarity between two compounds $d_{i}$ and $\;d_{j}$ can be calculated according to the Tanimoto coefficient as follows Eq. 2, and the chemical structure similarity matrix of the drug compound is expressed as $SIM_{strdrug}$ .

$$SIM_{strdrug_{ij}}=\frac{\left|d_{i\;}\cap \;d_{j}\right|}{\left|d_{i}\;\cup \;d_{j}\right|}$$ (2)

#### Genetic Data

Due to the rapid development of sequencing technology, a large amount of data has been accumulated, we use amino acid sequence data to measure the similarity of proteins. In this paper, normalized Smith-Waterman score (Smith and Waterman, 1981) is used to calculate the sequence similarity between protein $t_{i}$ and protein $t_{j}$ as shown in Eq. 3, where $SW\left(t_{i},\;t_{j}\right)$ represents the Smith-Waterman score between the target protein $t_{i}$ and $t_{j}$ , and the sequence similarity matrix of the protein is expressed as $SIM_{strtar}$ .

$$SIM_{strtar_{ij}}=\frac{SW\left(t_{i},\;t_{j}\right)}{\sqrt{SW\left(t_{i},\;t_{i}\right)SW\left(t_{j},\;t_{j}\right)}}$$ (3)

#### Drug-Target Interactions Data:

As for the performance evaluation of the DTIs prediction algorithm, we will verify it on four benchmark datasets, including E, IC, GPCR, and NR. Get the address of datasets:http://web.kuicr.kyoto-u.ac.jp/supp/yoshi/drugtarget/. Table 1 counts the relevant information of the four datasets, including the number of drugs (n), the number of targets (m), the number of interactions.

**TABLE 1:** Information about the four datasets.

Dataset	E	IC	GPCR	NR
Drugs(n)	445	210	223	54
Targets(m)	664	204	95	26
Interactions	2926	1476	635	90

According to the above $Y_{train}$ ∈ $R^{m\;\times \;n}$ , we use cosine similarity and Jaccard similarity coefficient to calculate the drug-drug similarity matrices $SIM_{cosdrug}$ , $SIM_{Jacdrug}$ , target-target similarity matrices $SIM_{costar}$ , $SIM_{Jactar}$ .

$$SIM_{cosdrug_{ij}}=\frac{\left|d_{i\;}\cap d_{j}\right|}{\sqrt{\left|d_{i}\right|\;\left|d_{j}\right|}}$$ (4)

$$SIM_{Jacdrug_{ij}}=\frac{\left|d_{i\;}\cap d_{j}\right|}{\left|d_{i}\cup d_{j}\right|}$$ (5)

$$SIM_{costar_{ij}}=\frac{\left|t_{i\;}\cap t_{j}\right|}{\sqrt{\left|t_{i}\right|\left|t_{j}\right|}}$$ (6)

$$SIM_{Jactar_{ij}}=\frac{\left|t_{i\;}\cap t_{j}\right|}{\left|t_{i\;}\cup t_{j}\right|}$$ (7)

Table 2 lists the above similarity matrices. There are three similarity matrices in the drug space and the target space respectively.

**TABLE 2:** Summary of similarity matrix of two feature spaces.

Space	Similarity matrix	Description
Drug	$SIM_{strdrug}$	Chemical structure
	$SIM_{cosdrug}$	Cosine similarity of drugs
	$SIM_{Jacdrug}$	Jaccard similarity of drugs
Target	$SIM_{strtar}$	Target sequence information
	$SIM_{costar}$	Cosine similarity of target
	$SIM_{Jactar}$	Jaccard similarity of the target

### NRLMF

NRLMF is the method proposed by Lin et al. (Liu et al., 2016), which combines Logistic matrix decomposition (LMF) and domain regularization to predict associations. Some of the equations that will be used in the model are as follows:

$$\begin{array}{l} \begin{array}{c} min\\ U,V \end{array}\sum_{i=1}^{m}\sum_{j=1}^{n}\left(1+cy_{ij}-\;y_{ij}\right)\ln\left[\;1+\exp\left(u_{i}V_{j}^{T}\right)\right]-cy_{ij}u_{i}V_{j}^{T}\\ +\frac{1}{2}\text{tr}\left[U^{T}\left(\lambda_{d}I+\alpha L^{d}\right)U\right]+\frac{1}{2}\mathrm{tr}\left[V^{T}\left(\lambda_{t}I+\beta L^{t}\right)V\right] \end{array}$$ (8)

Denoting the objective function in Eq. 8 by L, the partial gradients with respect to U and V are as follows:

$$\frac{\partial L}{\partial U}=\mathrm{PV}+\left(c-1\right)\left(Y⊙P\right)V-c\mathrm{YV}+\left(\lambda_{d}I+\alpha L^{d}\right)U$$ (9)

$$\frac{\partial L}{\partial V}=P^{T}U+\left(c-1\right)\left(Y^{T}⊙P^{T}\right)U-cY^{T}U+\left(\lambda_{t}I+\beta L^{t}\right)V$$ (10)

where **P**∈ $R^{m\;\times \;n}$ , in which the (I, j) element is $P_{ij}$ , ⊙ denotes the Hadamard product of two matrices. View reference Lin et al. (Liu et al., 2016), where we can see more details about above equation.

### Q-Learning Algorithm

**FIGURE 1:**
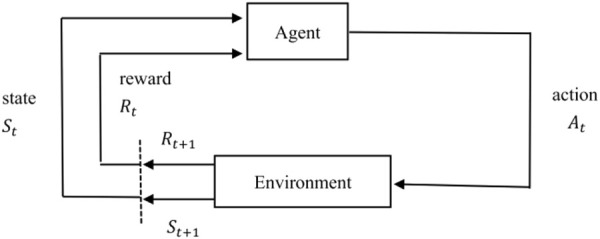
Reinforcement learning.

Reinforcement learning is an algorithm model that can make the best strategy through self-study in a particular situation. As shown in Figure 1, for real-world problems, reinforcement learning can be modeled by abstracting them as the interaction process between agent and environment. For each time step, the agent receives the state of the environment and chooses to perform the corresponding action, and then in the next time step, the agent obtains a reward and a new state based on the feedback of the environment. In other words, reinforcement learning refers to continuously learning to adapt to the environment according to the rewards obtained, and the goal of the agent is to maximize the expected cumulative reward.

Q-learning is a value-based algorithm among reinforcement learning algorithms. Q (*s*, *a*) is the expectation that taking action *a* (*a*∈A) can obtain benefits under state *s* (*s*∈S) at a certain moment, and the environment will feedback corresponding return *r* according to the action taken by the agent. Therefore, the main idea of this algorithm is to construct a Q-Table (as shown in Table 3) between state S and action A to store Q value, and then select the action that can obtain the maximum benefit according to Q value.

**TABLE 3:** Q-Table.

Q-Table	$a_{1}$	$a_{2}$
$s_{1}$	q ( $s_{1},\;a_{1}$ )	q ( $s_{1},\;a_{2}$ )
$s_{2}$	q ( $s_{2},\;a_{1}$ )	q ( $s_{2},\;a_{2}$ )
$s_{3}$	q ( $s_{3},\;a_{1}$ )	q ( $s_{3},\;a_{2}$ )

The updated formula for Q value calculation in Q-Table is as follows:

$$Q\left(s,a\right)\leftarrow Q\left(s,a\right)+\alpha \left[r+\gamma \max_{a^{\prime }}Q\left(s^{\prime },a^{\prime }\right)-Q\left(s,a\right)\right]$$ (11)

Where α is learning rate and γ is discount rate.

### MDP Modeling

#### State Space Design

Before the experiment begins, we initialize a set of weights for the drug similarity matrix and the target similarity matrix respectively, and then find a set of optimal weight assignments through the Q-learning algorithm. The state space at time step t is defined as $S_{t}$ = ( $\alpha_{t},\;\beta_{t},\;\gamma_{t},x_{t},\;y_{t},\;z_{t}$ ), where $\alpha_{t}$ , $\beta_{t}$ , $\gamma_{t}$ , $x_{t}$ , $y_{t}$ and $z_{t}$ represent respectively the weights in front of the three drug similarity matrices and the three target similarity matrices at the time step t, and $\alpha_{t}$ + $\beta_{t}$ + $\gamma_{t}$ = 1, $x_{t}$ + $y_{t}$ + $z_{t}$ = 1.

$$SIM_{drug}=\alpha SIM_{strdrug}+\beta SIM_{cosdrug}+\gamma SIM_{Jacdrug}$$ (12)

$$SIM_{tar}=xSIM_{strtar}+ySIM_{costar}+zSIM_{Jactar}$$ (13)

#### Action Space Design

In order to optimize the weight of the drug similarity matrix and the target similarity matrix, we define the action space as A = (a, b, c, -(b + c)), where a∈{0, 1}, 0 means that the current action only acts on the weight of the drug similarity matrix, and 1 means that it only acts on the weight of the target similarity matrix; b, c∈{−0.1, 0, 0.1}, for example, the current state is (0.3, 0.3, 0.4, 0.3, 0.3, 0.4) and the executed action is (0, 0.1, 0.1, −0.2), so the next state is (0.4, 0.4, 0.2, 0.3, 0.3, 0.4).

#### Design of Reward Function

After each action is performed, environment will respond with an instant reward *R* in return. In this paper, we will use AUC as a reward. When we perform an action *a* at each time step, we will get a new state *s* that is a new weight. Then, data integration is carried out according to the new weights, and the integration results and drug-target interactions data are taken as the input of the NRLMF model, and the output result AUC is used as the instant reward after performing the corresponding action. If the state does not match after the action is taken, that is, there is a situation that is less than 0 or greater than 1 in the state, we will feedback 0 as an instant reward.

$$R\left(S_{t}\right)=\{\begin{array}{cc} AUC & If\;all\;weights\;are\;between\;0\;and\;1\\ 0 & Otherwise \end{array}$$ (14)

### QLNRLMF

In our experiment, we divide the dataset into training set and test set in a ratio of 9:1, and validate the performance with ten-fold-cross-validation. The main ideas of our method are as follows: First, we use different similarity calculation methods to calculate heterogeneous data sources and obtain drug Space and target Space. Then, we initialize weights for the matrices in Drug space and target space respectively and optimize them through Q-learning algorithm. After obtaining the optimal weight, linear weighted integration is carried out to obtain $SIM_{drug}$ and $SIM_{tar}$ . Finally, we take $SIM_{drug}$ , $SIM_{tar}$ and $Y_{train}$ as the inputs of NRLMF model for DTIs prediction.

**FIGURE 2:**
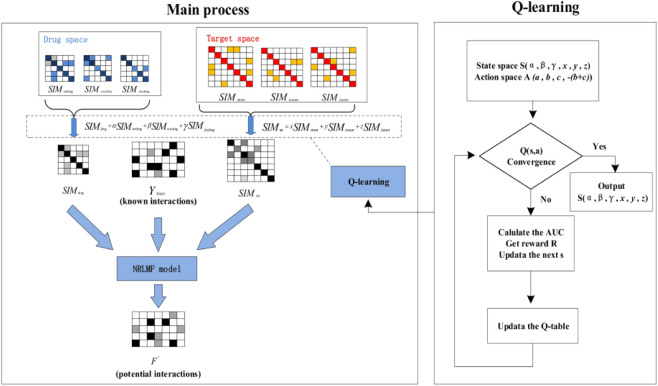
Algorithm flow chart.

The pseudocode of QLNRLMF algorithm is shown in Algorithm 1. The overall flow chart for QLNRLM is shown in Figure 2.

Algorithm 1 Qlnrlmf

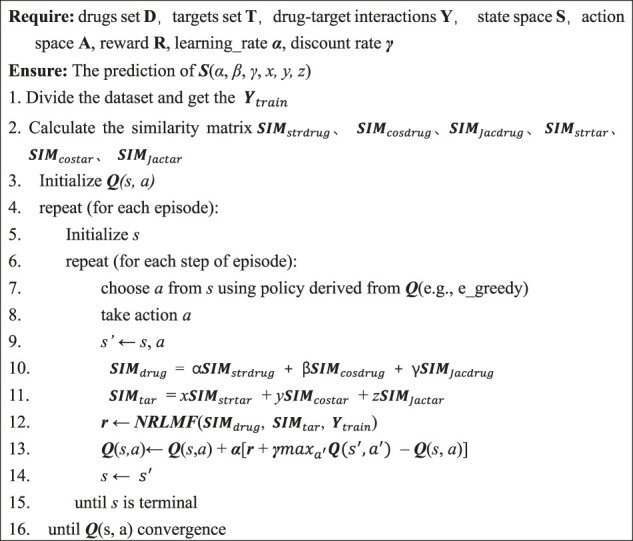

## Results

### Evaluation Measurements

In order to verify the performance of our proposed method, we evaluate it from the feasibility, efficiency and accuracy of the algorithm. First of all, we prove the feasibility of our algorithm through the convergence graph of the average reward for each episode. Secondly, we compare the Q-learning algorithm with the brute force method in terms of the time required to find the optimal weights, to prove the effectiveness of our algorithm. Finally, we compare our algorithm with other algorithms on AUC and AUPR to prove the accuracy of our algorithm.

### Average Reward Convergence Graph

**FIGURE 3:**
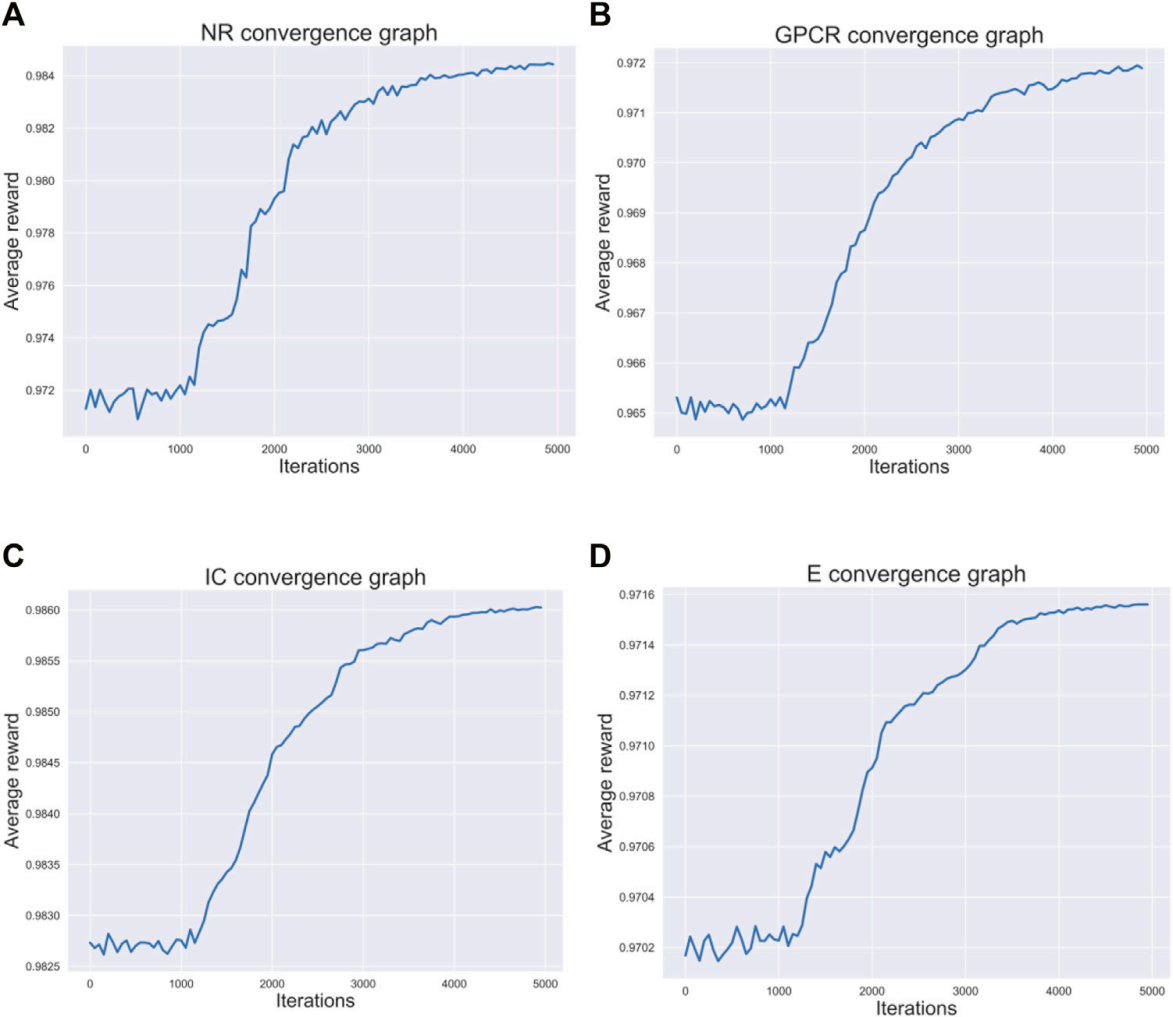
Four datasets convergence graphs. **(A)** The convergence graph of NR dataset. **(B)** The convergence graph of GPCR dataset. **(C)** The convergence graph of IC dataset. **(D)** The convergence graph of E dataset.

Figure 3 respectively describes the average reward convergence graph of four benchmark datasets under q-Learning algorithm, where the abscissa represents the number of iterations and the ordinate represents the average reward. As shown in the Figure 3, the average reward does not show an obvious upward trend in the first 1000 iterations, because the agent is constantly exploring trial and error at the beginning. After that, with the continuous accumulation of experience, the strategies learned by agents are getting better and better, and the average reward also presents an upward trend. When the number of iterations reaches 4000, the average reward basically tends to be stable, indicating that agent has learned an optimal strategy.

### Comparisons with Brute Force Algorithm

In this experiment, the number $S_{num}$ of the state space is 1296. If the Brute Force algorithm is adopted, the optimal state can be found by traversing 1296 states, that is, the optimal weight allocation can be obtained. If we use the Q-learning algorithm, we separately record the number of different states visited when each fold finds the optimal weight. As shown in Table 4, we can conclude that the ratio of the time required by the four datasets to the time required by the Brute Force algorithm are: 78.4, 79.9, 78.9, 79.1%, and the average time ratio is 79.1%. All in all, the running time of Q-learning algorithm has been reduced by nearly 20.9% on average.

**TABLE 4:** Time contrast between Q-learning algorithm and Brute Force algorithm.

Dataset	1	2	3	4	5	6	7	8	9	10	Avg	Avg/ $S_{num}$
NR	1023	1001	1032	1027	1029	987	1008	1016	1035	1005	1016	0.784
GPCR	1033	1069	1001	1050	1015	1055	1050	1015	1050	1024	1036	0.799
IC	997	1021	1030	1034	1045	1034	1027	1012	1006	1017	1022	0.789
E	1045	994	1036	1039	1031	1001	1032	1064	982	1026	1025	0.791

### Comparisons with Other Methods

There are many evaluation indexes for DTIs prediction methods. Among them, AUC and AUPR are widely used. AUC is the area under the receiver operating characteristic (ROC) curve, which can also be understood as the probability that a positive sample and a negative sample are randomly given, and the prediction probability of the positive sample is greater than the probability of the prediction probability of the negative sample. The value of AUC can directly evaluate the performance of the DTIs prediction method, and the greater the value, the better. AUPR is the area under the curve of precision and recall rate, and is a quantitative measurement method that can determine the average separation degree between the predicted fraction of real interactions and the predicted fraction of real non-interactions (Peng et al., 2017). Relatively few interactions are known for DTIs prediction. Therefore, AUPR is a more effective evaluation indicator than AUC, because AUPR takes some measures to reduce the impact of predicted fake DTIs data on the highest ranking scores (Davis and Goadrich, 2006). Therefore, we use these two indicators to evaluate the performance of our proposed method.

**FIGURE 4:**
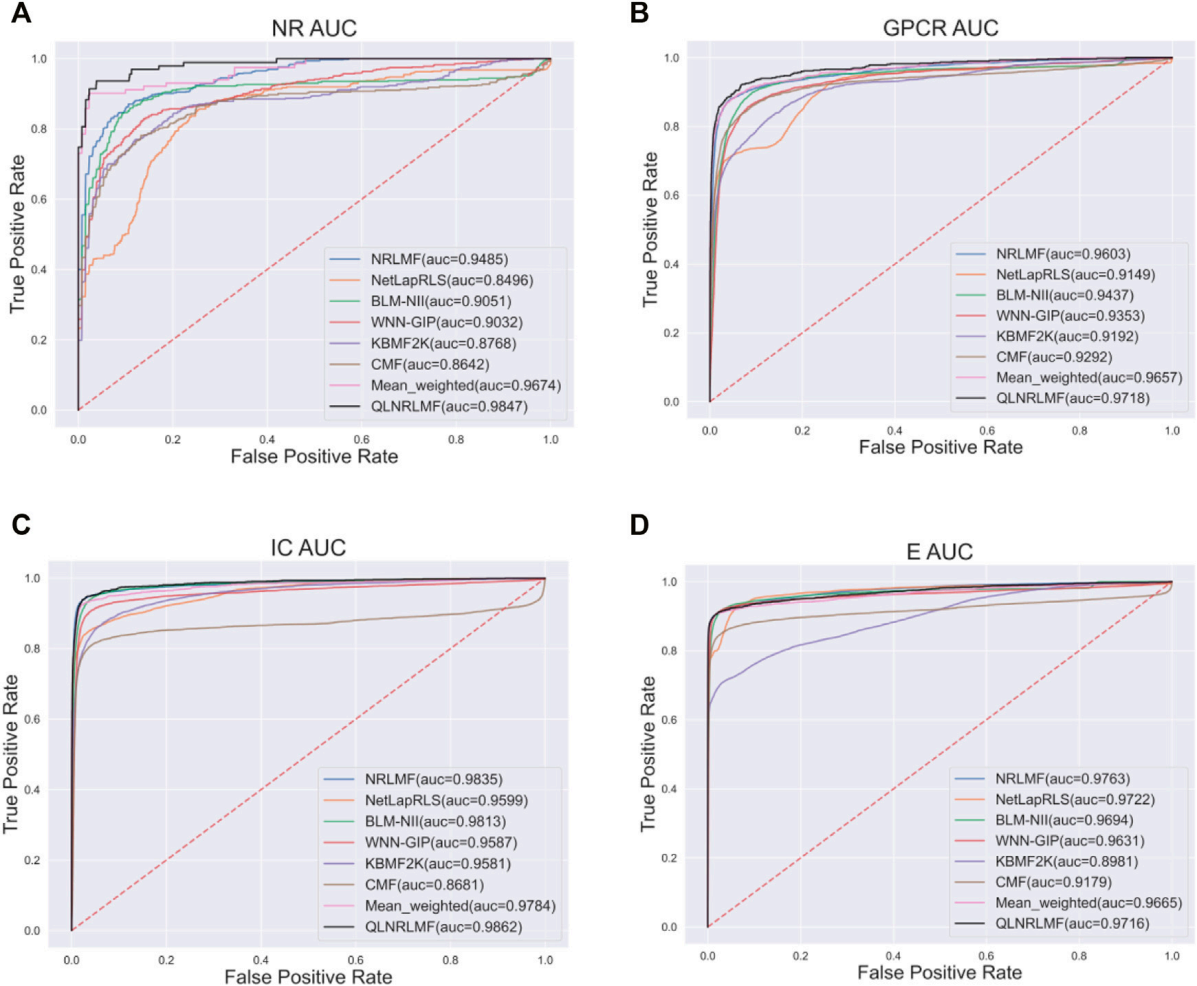
The AUC of QLNRLMF and Other Methods on Benchmark Datasets. **(A)** The AUC of QLNRLMF and Other Methods on NR Dataset. **(B)** The AUC of QLNRLMF and Other Methods on GPCR Dataset. **(C)** The AUC of QLNRLMF and Other Methods on IC Dataset. **(D)** The AUC of QLNRLMF and Other Methods on E Dataset.

**FIGURE 5:**
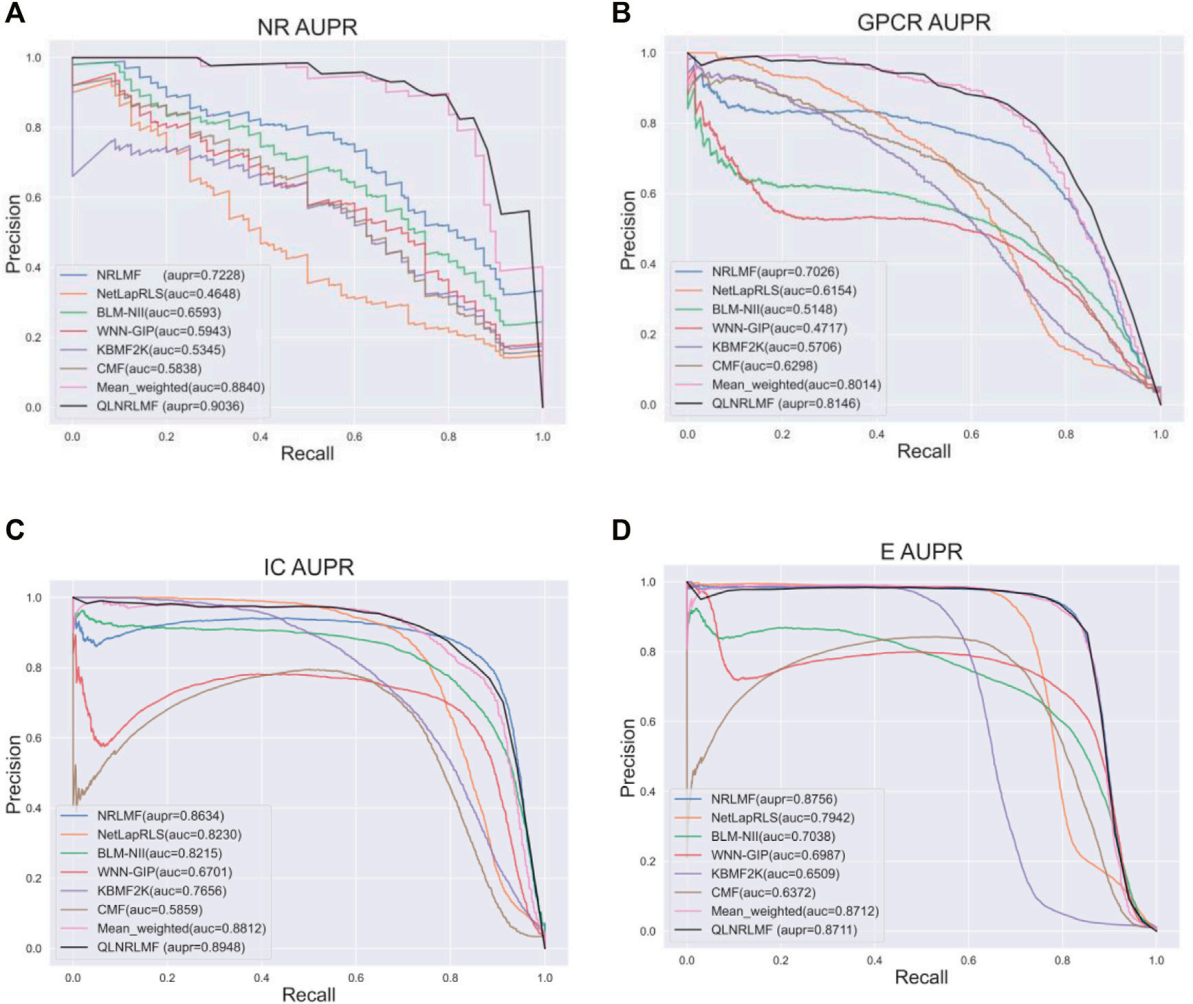
The AUPR of QLNRLMF and Other Methods on Benchmark Datasets. **(A)** The AUPR of QLNRLMF and Other Methods on NR Dataset. **(B)** The AUPR of QLNRLMF and Other Methods on GPCR Dataset. **(C)** The AUPR of QLNRLMF and Other Methods on IC Dataset. **(D)** The AUPR of QLNRLMF and Other Methods on E Dataset.

Figure 4; Figure 5 respectively describe the comparison of AUC and AUPR between our method and other seven methods under four benchmark datasets. As shown in the figures, the comparison between our QLNRLMF method and NRLMF method shows that in four datasets, our method improves 3.62, 1.15, 0.27% and -0.47% respectively in AUC, and 18.08, 11.23, 3.25% and −0.48% respectively in AUPR. In general, our method is superior to NRLMF method. In order to prove the advantages of Q-learning algorithm, we also perform the integration experiment of linear equal-weight strategy. The experimental results show that the linear integration strategy based on Q-learning algorithm is obviously better than the linear equal-weight integration strategy. In addition, we also compare our algorithm with other advanced five methods, which contain NetLapRLS (Xia et al., 2010), BLM-NII (Mei et al., 2013b), WNN-GIP (van Laarhoven and Marchiori, 2013), KBMF2K (Gönen, 2012) and CMF (Zheng et al., 2013), we can find that our proposed method has certain advantages from the figures. Finally, we summarize the experimental results and data of all the above methods into Table 5, from which we find that our QLNRLMF method achieves better results in DTIs prediction.

**TABLE 5:** Comparsion with the other seven methods.

AUC								
Dataset	NetLapRLS	BLM-NII	WNN-GIP	KBMF2K	CMF	Mean-weighted	NRLMF	QLNRLMF
NR	0.849	0.905	0.903	0.876	0.864	0.967	0.948	**0.984**
GPCR	0.914	0.943	0.935	0.919	0.929	0.965	0.960	**0.971**
IC	0.959	0.981	0.958	0.958	0.868	0.978	0.983	**0.986**
E	0.972	0.969	0.963	0.898	0.917	0.966	**0.976**	0.971
Avg	0.923	0.949	0.939	0.912	0.894	0.969	0.966	**0.978**
AUPR								
NR	0.464	0.659	0.594	0.534	0.583	0.884	0.722	**0.903**
GPCR	0.615	0.514	0.471	0.570	0.629	0.801	0.702	**0.814**
IC	0.823	0.821	0.670	0.765	0.585	0.881	0.863	**0.894**
E	0.794	0.703	0.698	0.650	0.637	0.871	**0.875**	0.871
Avg	0.674	0.674	0.608	0.629	0.608	0.859	0.790	**0.870**

The values in bold mean the best result for each line.

## Conclusion and Discussion

In this paper, we propose a model for optimizing weight allocation of heterogeneous data based on Q-learning algorithm to improve the accuracy of DTIs prediction. We obtain multiple drug-drug similarity matrices and target-target similarity matrices from heterogeneous data through different similarity calculation methods, and then optimize the linear weighted weights of these similarity matrices based on Q-learning algorithm. Finally, we perform the experiment of DTIs prediction based on NRLMF model. To evaluate the performance and advantages of our proposed QLNRLMF method, we perform a series of experiments on four benchmark datasets to demonstrate the feasibility, efficiency, and accuracy of our proposed method. There are two main advantages to our approach. In our study, we use AUC and AUPR as evaluation indicators to evaluate the performance of our proposed method. On the one hand, it can be seen from the experimental results that our method achieves better results on the four benchmark datasets compared with other methods. On the other hand, we add reinforcement learning method, which speeds up the acquisition of optimal weight and enables us to predict DTIs more effectively.

Through this experiment, we can find that the integration of multiple information can improve the prediction accuracy of DTIs to some extent, and the rational allocation of the weight of these information also plays a key role in the prediction performance of DTIs. One of our future work directions is to further propose better optimization algorithms for weight distribution. For example, we can change the action space from discrete to continuous to make the weight distribution more accurate, so as to further improve the accuracy of DTIs prediction.

## Data Availability

The original contributions presented in the study are included in the article/supplementary material, further inquiries can be directed to the corresponding authors.
